# 

**Published:** 1892-02-13

**Authors:** 


PRICE ONE PENNY.

No. 281, Vol. XI.—February 13th, 1892.

[Registered at the General Post Office as a Newspaper for transmission in the United Kingdom and Abroad.]

WITH EXTRA NURSING SUPPLEMENT.
Per Annum, 6s. 6d., post free.
Monthly Parts, 6d.; post free, 8d.
Ditto per Annum, 8s., post free

### THE HOSPITAL

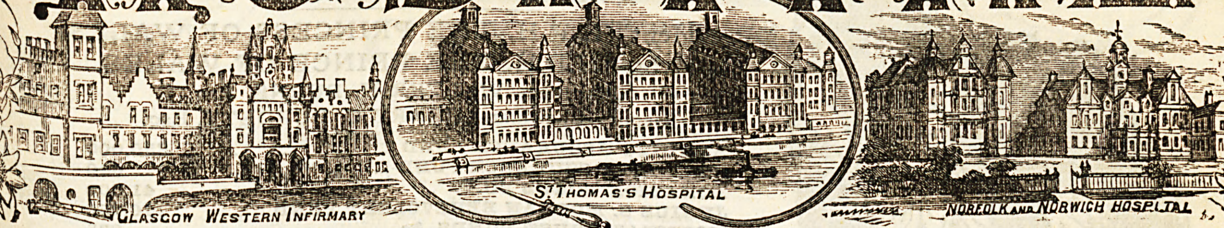

A WEEKLY JOURNAL OF

### Science, Medicine, Nursing, and Philanthropy.

### SATURDAY, FEBRUARY 13th, 1892.

**Death in the Church** - 237

**Passing Topics.**—Breakers Ahead - 238

**Some Scientific Aspects of Insanity.** XII.—General Paralysis - 239

**The Doctor's Armchair.**—The Farming of Patients - 240

**Everybody's Page** - 241

**Notes and News** - 242

**General Charities** - 243

**Annotations.**—A Veteran of the Sunday Fund—Is Influenza Infectious?—The Registration of Nurses - 244

**Scraps and Gleanings** - 243

**The Practitioner's Mirror and Retrospect**—

Surgery.—Congenital Displacement of the Hips - 245

Laryngology.—Intubation of the Larynx - 246

Inebriety and the Legislature - 247

New Drugs, Appliances, and Things Medical.—The Invigorator Corset - 247

**Some American Hospitals.** VII.—Pennsylvania Hospital 248

**The Student of Medicine.**—Appointments—Vacancies—Notes from the Universities—Pass Lists—Athletics.

### EXTRA SUPPLEMENT.—THE NURSING MIRROR.

**En Passant.**—A New Training Home—Homes for Nurses—Rate-Supported Hospitals—The Absent Nurse—Short Items—The Famine in Russia—Nightingale Notes - cxv
**Lectures on Surgical Ward Work and Nursing.** By Alex. Miles, M.D.Edin., F.R.C.S.E. Lecture XLIV.—Instruments for Mouth - cxvi
**Inasmuch—Presentation** - cxvii
**Wants and Workers** - cxvii
**For Reading to the Sick.**—Patience - cxvii
**Everybody's Opinion.**—The Sir Alexander Morison Prizes cxviii
**Notes from Australia** - cxviii
**Medical Women** - cxix
**Examination Questions** - cxix
**Notes and Queries** - cxix
**Off Duty.**—"Australian Papers, Please Copy" - cxx
**Where to Go** - cxx

